# Effect of a multimodal bundled intervention on quality of recovery after laparoscopic colorectal cancer surgery: a single-center, single-blind, pragmatic randomized controlled trial

**DOI:** 10.1186/s12957-026-04454-9

**Published:** 2026-06-17

**Authors:** Jiayi Ren, Qiuyu Yang, Haoxiang He, Jin Li, Hang Gao, Chen Yang, Yan Cao, Yuting Wen, Chen Chen, Marim Kandil, Jiming Tao

**Affiliations:** 1 Department of Rehabilitation, Shuguang Hospital, Shanghai University of Traditional Chinese Medicine, Shanghai, 203204 China; 2 Shanghai University of Traditional Chinese Medicine, Shanghai, 203204 China

**Keywords:** Multimodal bundled intervention, Colorectal cancer surgery, Randomized controlled trial, Electroacupuncture, Abdominal massage, Exercise therapy

## Abstract

**Background:**

Despite the minimally invasive nature of laparoscopic colorectal surgery, postoperative recovery is frequently compromised by pain, fatigue, and delayed gastrointestinal function. Current rehabilitation strategies largely rely on unimodal approaches, and evidence for structured multimodal bundled interventions is limited. This study aimed to evaluate whether a multimodal bundled intervention comprising electroacupuncture, abdominal massage, structured breathing training, and symptom-titrated supervised ambulation improves quality of recovery in patients undergoing laparoscopic surgery for stage I–III colorectal cancer.

**Methods:**

This single-center, single-blind, parallel-group, pragmatic randomized controlled trial randomized 105 patients to either a multimodal bundled intervention plus standard care or standard care alone. The intervention group received electroacupuncture and structured breathing training from postoperative day 1 to 7, with abdominal massage and symptom-titrated supervised ambulation added from postoperative day 4 to 7. The primary outcome was the trajectory of the Quality of Recovery-15 (QoR-15) score assessed at postoperative days 3, 7, and 30. Statistical analyses followed a modified intention-to-treat principle.

**Results:**

Of 105 randomized patients, 90 (45 per group) were included in the final analysis. A significant group-by-time interaction was observed for QoR-15 scores (*P* = 0.043). While no significant between-group differences were found at postoperative days 3 or 7, the intervention group demonstrated significantly higher QoR-15 scores at postoperative day 30 (mean difference 11.33; 95% confidence interval 3.41 to 19.26; *P* = 0.005). Secondary outcomes, including pain intensity and perceived exertion, showed favorable between-group differences at later postoperative assessments. No serious intervention-related adverse events were reported.

**Conclusions:**

In this single-center pragmatic randomized controlled trial, the multimodal bundled intervention did not significantly improve QoR-15 scores during the early postoperative period at POD3 or POD7. However, it was associated with a clinically meaningful improvement in QoR-15 at POD30. These findings suggest a potential medium-term patient-reported recovery benefit after laparoscopic colorectal cancer surgery, but they should not be interpreted as evidence of accelerated early ERAS-related recovery.

**Trial registration:**

ChiCTR2400085191. Registered on June 3, 2024.

**Supplementary Information:**

The online version contains supplementary material available at https://doi.org/10.1186/s12957-026-04454-9.

## Introduction

Colorectal cancer (CRC) remains one of the most prevalent malignancies worldwide [1]. While laparoscopic surgery has become the standard approach owing to its minimally invasive nature, the postoperative course is frequently complicated by acute pain, profound fatigue, impaired functional capacity, and delayed gastrointestinal motility [2, 3]. These distressing symptoms not only hinder early mobilization but also cumulatively compromise the patient’s overall Quality of Recovery (QoR), potentially adversely influencing long-term clinical outcomes.

Enhanced Recovery After Surgery (ERAS) protocols have substantially improved perioperative outcomes by integrating evidence-based strategies, including multimodal analgesia, early mobilization, and optimized perioperative care [4]. Recent evidence underscores that effective ERAS pathways not only improve short-term clinical outcomes but may also facilitate timely initiation of subsequent oncological treatments [5, 6]. However, standardized ERAS pathways may be difficult to implement uniformly in heterogeneous surgical oncology populations, particularly among older or frail patients who may have limited tolerance for fixed early mobilization targets [7, 8]. Conventional postoperative ambulation protocols often rely on general encouragement or “one-size-fits-all” instructions, with limited use of symptom-titrated strategies to guide safe activity progression. In this context, non-pharmacological modalities that may reduce pain, fatigue, and gastrointestinal discomfort could potentially support early rehabilitation within ERAS pathways.

In recent years, complementary and integrative approaches, including acupuncture-related therapies and abdominal massage, have gained increasing attention in perioperative settings for alleviating postoperative symptoms and supporting recovery [9]. Furthermore, techniques such as transcutaneous electrical acupoint stimulation (TEAS) have shown potential for reducing postoperative pain and facilitating gastrointestinal recovery after abdominal surgery [10]. Building upon this emerging evidence, structured multimodal bundled interventions may help address these limitations by simultaneously targeting multiple physiological and symptomatic pathways [11, 12]. Electroacupuncture may exert analgesic and anti-inflammatory effects and may influence gastrointestinal motility through autonomic regulation [13–15]. Abdominal massage may also support gastrointestinal function by stimulating local circulation and innulls testinal peristalsis [16, 17]. When combined with structured breathing training and symptom-titrated supervised ambulation, these components may constitute a clinically feasible multimodal bundled intervention targeting several barriers to postoperative recovery.

Despite this biologically plausible rationale, high-quality randomized controlled trials evaluating coordinated multimodal bundled interventions after laparoscopic colorectal cancer surgery remain limited. Because the intervention components are intended to be delivered together as a real-world care bundle, isolating the efficacy of each modality against sham controls may not fully reflect pragmatic perioperative practice. Therefore, we conducted a single-center, single-blind pragmatic randomized controlled trial to evaluate whether a multimodal bundled intervention, comprising electroacupuncture, abdominal massage, structured breathing training, and symptom-titrated supervised ambulation, could improve the postoperative QoR-15 trajectory in patients undergoing laparoscopic surgery for stage I–III colorectal cancer compared with standard postoperative care alone.

## Methods

### Study design

This single-center, single-blind pragmatic randomized controlled trial was conducted at Shuguang Hospital Affiliated to Shanghai University of Traditional Chinese Medicine. The trial was designed to evaluate the effectiveness of a multimodal bundled intervention under real-world postoperative care conditions. The study was approved by the Ethics Committee of Shuguang Hospital (Approval No. 2024–1494-077–01) and was prospectively registered at the Chinese Clinical Trial Registry (ChiCTR2400085191). All participants provided written informed consent before enrollment.

### Participants

Patients aged 18–80 years who underwent elective laparoscopic resection for stage I–III colon or upper rectal cancer and were able to read and communicate in Chinese were considered eligible. Key exclusion criteria included mid/low rectal cancer, creation of a stoma, conversion to open surgery, pacemaker implantation, allergy to acupuncture needles, or prior experience with acupuncture interventions. Patients with metastatic disease, ASA physical status > III, or severe cardiac failure were also excluded. Additional baseline clinical variables, including ASA physical status, AJCC tumor stage, tumor location, surgical procedure, and major comorbidities, were retrospectively extracted from medical records to better characterize baseline comparability.

### Randomization and blinding

An independent statistician generated the randomization sequence using a computer-based random number generator in SPSS version 26.0. Participants were allocated in a 1:1 ratio to the multimodal bundled intervention group or the standard postoperative care group. Stratified block randomization was employed to ensure balance across key baseline variables, including AJCC tumor stage, age, and sex. Allocation concealment was maintained using sequentially numbered, opaque, sealed envelopes (SNOSE). After baseline assessment and written informed consent, a research nurse who was not involved in enrollment or outcome assessment opened the envelope and assigned participants to the allocated group.

Because of the behavioral and procedural nature of the intervention, blinding of participants and therapists was not feasible. To reduce performance and detection bias, participants were informed that the study compared two postoperative recovery approaches, without emphasizing the expected superiority of either group, and were instructed not to discuss intervention details with outcome assessors. Routine surgical and ward care was delivered according to standardized institutional protocols, and staff involved in routine care were not involved in outcome assessment.

Outcome assessors and the data analyst remained blinded to group allocation throughout the study. All outcomes were assessed at prespecified time points by trained blinded personnel. Intervention-related adverse events were managed by unblinded therapists according to protocol without unblinding the outcome assessors. These procedures were implemented to minimize selection, performance, and detection bias.

### Interventions

Participants were randomly assigned to either the multimodal bundled intervention group plus standard postoperative care or the control group (standard postoperative care alone). The intervention consisted of three modules: electroacupuncture, abdominal massage, and exercise therapy (structured breathing training and symptom-titrated supervised ambulation). The components and procedures of each group are summarized in Table 1, and the day-by-day intervention schedule across postoperative days 1–7 (POD1–POD7) is provided in Table 2.

**Table 1:** Intervention protocol details

Component	Intervention Group (Multimodal bundled intervention)	Control Group (Standard Postoperative Care)
Basis of Care	Received all components of standard postoperative care based on institutional ERAS protocols, plus the following bundled intervention modules	Received standard postoperative care only(based on institutional ERAS protocols)
Electroacupuncture		NA
› Practitioner	Licensed acupuncturists with ≥ 5 years of experience	
› Acupoints	Bilateral Zusanli (ST-36), Hegu (LI-4)	
› Needle Specification	Sterile, single-use needles (0.40 mm × 35 mm; Qizhou, Suzhou Zhongjing Life Technology Co., Ltd., China)	
› Depth/Technique	~ 20–30 mm depth; de qi sensation sought	
› Electroacupuncture Device	Hwato SDZ-III electronic acupuncture device (Suzhou Medical Appliance Factory, China)	NA
› Waveform/Frequency	Continuous wave, 100 Hz	
› Session Duration & Frequency	20 min, once daily	
› Period	POD1–POD7 (Total: 7 sessions)	
Abdominal Massage (Tuina)		NA
› Practitioner	Certified physiotherapists trained in Tuina-based abdominal massage	
› Technique	Gentle clockwise abdominal rubbing (Mo Fa)	
› Pressure	Light pressure, ensuring no pain	
› Session Duration & Frequency	10 min, twice daily	
› Period	POD4–POD7	
Exercise Therapy		
› Breathing Training	Diaphragmatic breathing, 5 min, twice daily	No structured breathing training. General verbal encouragement for early mobilization was provided
› Ambulation Plan	POD4–POD7: Symptom-titrated supervised progressive ambulation, twice daily • Target walking duration: 5–15 min per session, adjusted according to predefined safety thresholds and patient tolerance • Titration criteria: Modified Borg score ≤ 3 and VAS pain score ≤ 4	No structured ambulation plan

**Table 2:** Intervention schedule

Postoperative Days (POD)	Electroacupuncture	Breathing Training	Abdominal Massage (Tuina)	Early Ambulation
POD 1–3	✔ Once daily; 20 min/session	✔Twice daily; 5 min/session	-	-
POD 4–7	✔ Once daily; 20 min/session	✔ Twice daily; 5 min/session	✔ Twice daily; 10 min/session	✔ Twice daily; target 5–15 min/session

#### Intervention group (multimodal bundled intervention)

The intervention group received a multimodal bundled intervention in addition to standard postoperative care, including:

1. Electroacupuncture: Electroacupuncture was performed by licensed acupuncturists at predefined acupoints, including Zusanli (ST-36) and Hegu (LI-4). Electrical stimulation was applied at a tolerable intensity using a continuous waveform of 100 Hz. Each session lasted 20 min and was delivered once daily from POD1 to POD7.
2. Abdominal Massage: Abdominal massage was performed by certified physiotherapists using gentle clockwise abdominal rubbing, namely the Mo Fa technique. Each session lasted 10 min and was administered twice daily from POD4 to POD7.
3. Exercise Therapy: Exercise therapy included structured breathing training and symptom-titrated supervised ambulation. Structured breathing training was led by certified physiotherapists according to a standardized stepwise protocol and was performed for 5 min per session, twice daily from POD1 to POD7. Symptom-titrated supervised ambulation was performed twice daily from POD4 to POD7. Walking duration was adjusted according to predefined safety thresholds, including a Modified Borg score ≤ 3 and VAS pain score ≤ 4. Ambulation sessions were paused or maintained at the previously tolerated level if these thresholds were exceeded.

Detailed intervention parameters, including personnel, procedures, session duration, frequency, and safety criteria, are provided in Table 1.

#### Control group (standard postoperative care)

Patients in the control group received standard postoperative care based on institutional ERAS protocols, including standardized pharmacologic pain management, routine vital signs monitoring, fluid management, and general verbal encouragement for early mobilization. They did not receive electroacupuncture, abdominal massage, structured breathing training, or the symptom-titrated supervised ambulation protocol provided to the intervention group. All patients in both groups received a standardized multimodal analgesia protocol according to institutional ERAS guidelines to minimize potential confounding from differences in pain management.

### Outcome measures

#### Primary outcome

The primary outcome was the patient-centred Quality of Recovery-15 (QoR-15) score. The validated Chinese version of the QoR-15 was used because all participants could read and communicate in Chinese [18]. QoR-15 was selected because it is a concise, validated short-form recovery measure derived from QoR-40 and is suitable for repeated postoperative assessments with lower respondent burden [19]. The QoR-15 includes 15 items covering five domains: pain, physical comfort, physical independence, psychological support, and emotional state. Total scores range from 0 to 150, with higher scores indicating better recovery. A score of ≥ 118 [20] was defined as indicating good recovery, and the minimal clinically important difference (MCID) was set at 6.0 [21]. QoR-15 scores were assessed on postoperative day 3 (POD3), day 7 (POD7), and day 30 (POD30) to capture short- to medium-term recovery dynamics following laparoscopic colorectal cancer surgery. The QoR-15 is widely recognized as a patient-reported outcome (PRO) tool in clinical recovery assessments.

#### Secondary outcomes

Secondary outcomes included pain intensity, muscle strength, perceived exertion, and functional exercise capacity. Pain intensity was assessed using the Visual Analog Scale (VAS; 0–10). Muscle strength was evaluated using the Medical Research Council (MRC) scale (0–5), where 0 indicates no visible or palpable muscle contraction and 5 indicates normal strength. Assessments were performed by trained assessors according to a standardized protocol on postoperative days (POD) 3, 7, and 30. Perceived exertion was evaluated using the Modified Borg Scale (0–10), and functional capacity was objectively assessed using the 6-min walk test (6MWT). As early mobilization may be restricted in the immediate postoperative period, formal assessments of perceived exertion (Borg) and functional capacity (6MWT) were conducted on POD7 and POD30. (Note: The real-time, daily VAS and Borg scores utilized exclusively for the titration of the early ambulation plan on POD 4–7 were recorded in nursing charts for safety monitoring and adherence tracking, distinct from these formal secondary outcome assessments).

### Sample size calculation

The sample size calculation was based on the primary outcome, the QoR-15 score on postoperative day 3. Although the primary analysis assessed the overall recovery trajectory, the sample size was conservatively powered based on the early critical time point of POD3. POD3 was selected because it reflects early global recovery beyond the immediate post-anesthesia period and has been used as a primary time point in perioperative recovery trials employing QoR-15 [22]. Based on published QoR-15 data in laparoscopic colorectal surgery and related recovery studies, together with our clinical experience, the expected mean (SD) POD3 QoR-15 score in the control group was 110 [12], based on published data and clinical experience [23, 24]. A between-group difference of 6.0 points was considered clinically meaningful (MCID) [19–21], corresponding to a standardized effect size (Cohen’s d) of 0.50. Using a two-sided independent-samples t-test with an α level of 0.05, 80% power, and a 1:1 allocation ratio, G*Power software (version 3.1.9.7) indicated that 36 participants were required per group. To achieve a final evaluable sample of 90 participants (45 per group) after accounting for potential post-randomization exclusions and dropouts, we ultimately randomized a total of 105 patients. This final evaluable sample size met the prespecified target sample size after accounting for potential post-randomization exclusions and dropouts.

### Statistical analysis

All analyses were conducted according to a modified intention-to-treat (mITT) principle, including all randomized participants with available outcome data. Descriptive statistics were utilized to summarize baseline characteristics, adverse events, and adherence to the progressive ambulation protocol (e.g., achievement of target duration and protocol interruptions). All statistical analyses were performed using SPSS version 24.0. Continuous variables were assessed for normality using the Shapiro–Wilk test. Data are presented as mean ± standard deviation for normally distributed variables or median (interquartile range) for non-normally distributed variables. Categorical variables are presented as frequencies (percentages) and were compared using chi-square tests. Baseline characteristics were compared between groups using independent-samples t tests or Mann–Whitney U tests for continuous variables, and χ^2^ tests or Fisher’s exact tests for categorical variables, as appropriate.

For longitudinal outcomes, including QoR-15 scores, postoperative pain intensity (VAS), muscle strength, Borg Rating of Perceived Exertion scores, 6-min walk test (6MWT) distance, and the percentage of predicted 6MWT distance, linear mixed-effects models were used to account for within-subject correlations over time. Each model included fixed effects for group (intervention vs control), time, and their interaction, along with a subject-specific random intercept to account for within-subject correlations. This likelihood-based approach naturally handles missing data under the missing-at-random (MAR) assumption. When a significant group-by-time interaction was detected, between-group comparisons were performed at each time point using estimated marginal means. In the absence of a significant interaction, main effects of group and time were interpreted. Post hoc pairwise comparisons were adjusted for multiple testing using the Bonferroni method. Results from mixed-effects models are reported as estimated marginal means with standard errors (SE), along with mean differences, 95% confidence intervals (CI), and corresponding *P* values. All statistical tests were two-sided, and a P value < 0.05 was considered statistically significant.

### Results

#### Participant flow

**Fig. 1:**
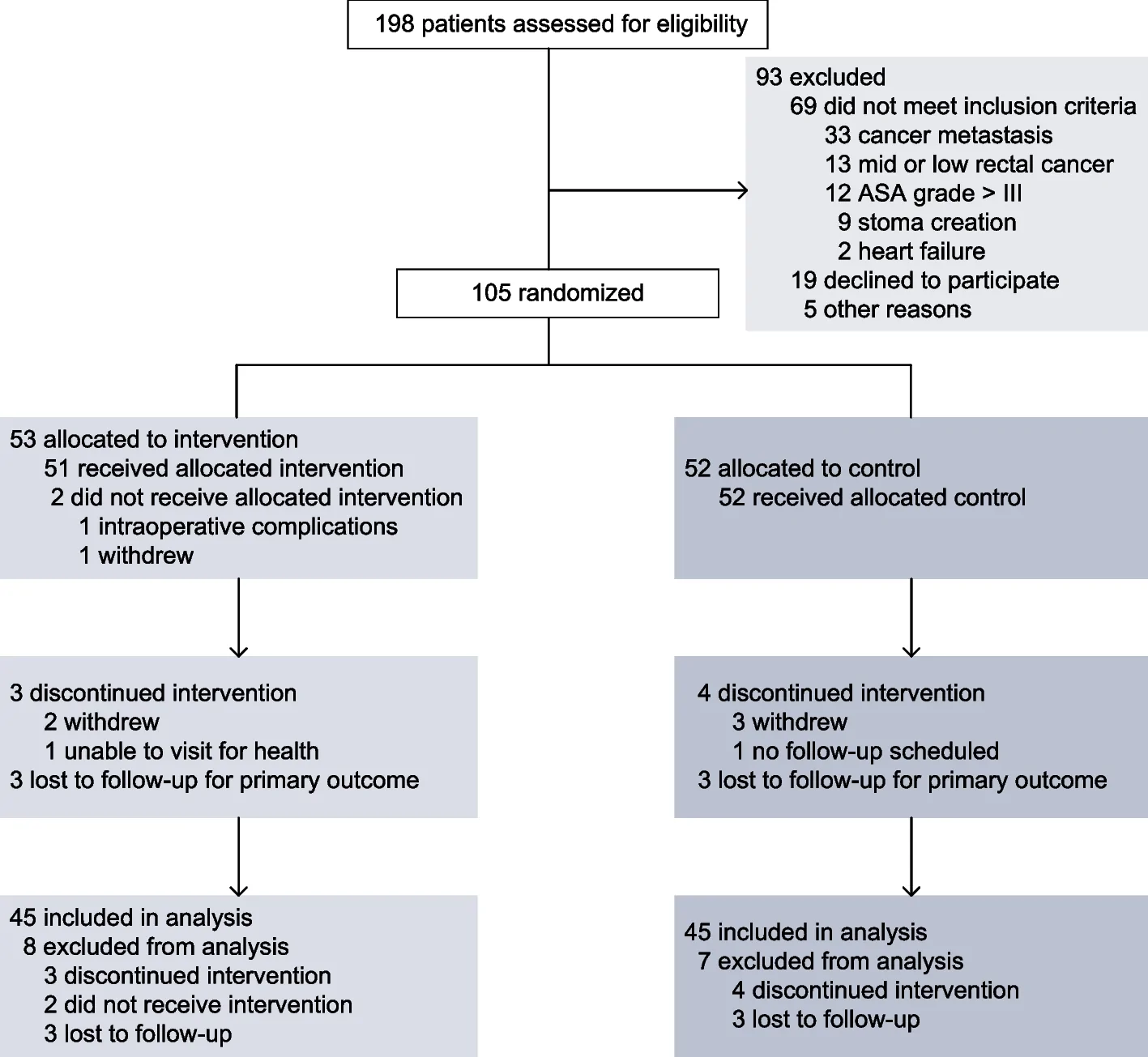
CONSORT Flow Diagram

A total of 198 patients were assessed for eligibility, of whom 105 were randomized to the intervention group (*n* = 53) or the control group (*n* = 52). After randomization, two participants in the intervention group did not receive the allocated intervention due to intraoperative complications (*n* = 1) or withdrawal of consent (*n* = 1). During follow-up, six participants in the intervention group and seven in the control group discontinued the intervention or were lost to follow-up for the primary outcome. Ultimately, 45 participants in each group had at least one post-baseline assessment of the primary outcome and were included in the mITT analysis. The detailed reasons for exclusion after randomization are presented in the CONSORT flow diagram (Fig. 1).

### Baseline, oncological, and surgical characteristics

Baseline demographic, oncological, surgical, and preoperative clinical characteristics were generally well balanced between the two groups (Table 3). No statistically significant differences were observed in demographic variables, ASA physical status, AJCC tumor stage, tumor location, surgical procedure, major comorbidities, preoperative hemoglobin or albumin levels, neoadjuvant therapy, previous abdominal surgery, operative time, or estimated blood loss (all *P* > 0.05). These findings indicate that randomization achieved comparable baseline and perioperative characteristics between the groups.

**Table 3:** Baseline demographic, oncological, surgical, and preoperative clinical characteristics of participants

Characteristic	Control group (*n* = 45)	Intervention group (*n* = 45)	*P* value
Age, y	70 (61, 78)	72 (61, 80)	0.401
Sex, n (%)			0.672
Female	19 (42.2)	22 (48.9)	
Male	26 (57.8)	23 (51.1)	
Height, cm	166 (162, 171)	166 (158, 172)	0.665
Weight, kg	64.7 ± 10.9	65.9 ± 11.3	0.615
Body mass index, kg/m^2^	23.4 ± 3.2	24.3 ± 3.9	0.256
ASA physical status, n (%)			0.666
I	29 (64.4)	26 (57.8)	
II	16 (35.6)	19 (42.2)	
III	0 (0.0)	0 (0.0)	
AJCC tumor stage, n (%)			0.283
I	13 (28.9)	11 (24.4)	
II	20 (44.4)	27 (60.0)	
III	12 (26.7)	7 (15.6)	
Tumor location, n (%)			1.000
Colon	42 (93.3)	43 (95.6)	
Upper rectum	3 (6.7)	2 (4.4)	
Surgical procedure, n (%)			0.642
Laparoscopic right hemicolectomy	7 (15.6)	6 (13.3)	
Laparoscopic left hemicolectomy	17 (37.8)	23 (51.1)	
Laparoscopic sigmoidectomy	18 (40.0)	14 (31.1)	
Laparoscopic anterior resection	3 (6.7)	2 (4.4)	
Major comorbidities, n (%)			
Hypertension	16 (35.6)	16 (35.6)	1.000
Diabetes mellitus	9 (20.0)	7 (15.6)	0.784
Cardiovascular disease	8 (17.8)	4 (8.9)	0.353
Preoperative hemoglobin, g/L	113.8 ± 12.7	117.7 ± 13.5	0.160
Preoperative albumin, g/L	41 (37, 46)	41 (36, 45)	0.230
Neoadjuvant therapy, n (%)	3 (6.7)	2 (4.4)	1.000
Previous abdominal surgery, n (%)	4 (8.9)	6 (13.3)	0.739
Operative time, min	176.6 ± 25.4	174.2 ± 25.9	0.667
Estimated blood loss, mL	80 (75, 105)	75 (65, 95)	0.103

### Intervention adherence and safety

The multimodal bundled intervention, particularly the symptom-titrated progressive ambulation protocol, was well tolerated. By POD 7, 38 patients (84.4%) in the intervention group successfully achieved the target walking duration of 10–15 min per session. Protocol interruptions or failure to progress due to predefined intolerance criteria occurred in 7 patients. The primary reasons for limiting walking duration were postoperative wound pain (VAS > 4, *n* = 3) and intolerable fatigue (Modified Borg > 3, *n* = 4). Notably, all these patients safely maintained their previously tolerated durations. No serious intervention-related adverse events (e.g., severe bleeding, falls, or anastomotic leakage) were reported in either group. A few minor incidents, such as transient mild bruising at the acupuncture sites or mild muscle soreness after massage, were noted but resolved spontaneously without requiring medical intervention or protocol discontinuation.

### Primary outcome

#### Quality of recovery-15 (QoR-15) scores

Quality of recovery was assessed using the QoR-15 questionnaire at POD3, POD7, and POD30. Linear mixed-effects model analysis demonstrated a significant group-by-time interaction (*P* = 0.043), indicating different postoperative recovery trajectories between the two groups. A significant main effect of time was observed (*P* < 0.001), whereas the main effect of group did not reach statistical significance (*P* = 0.053).

**Fig. 2:**
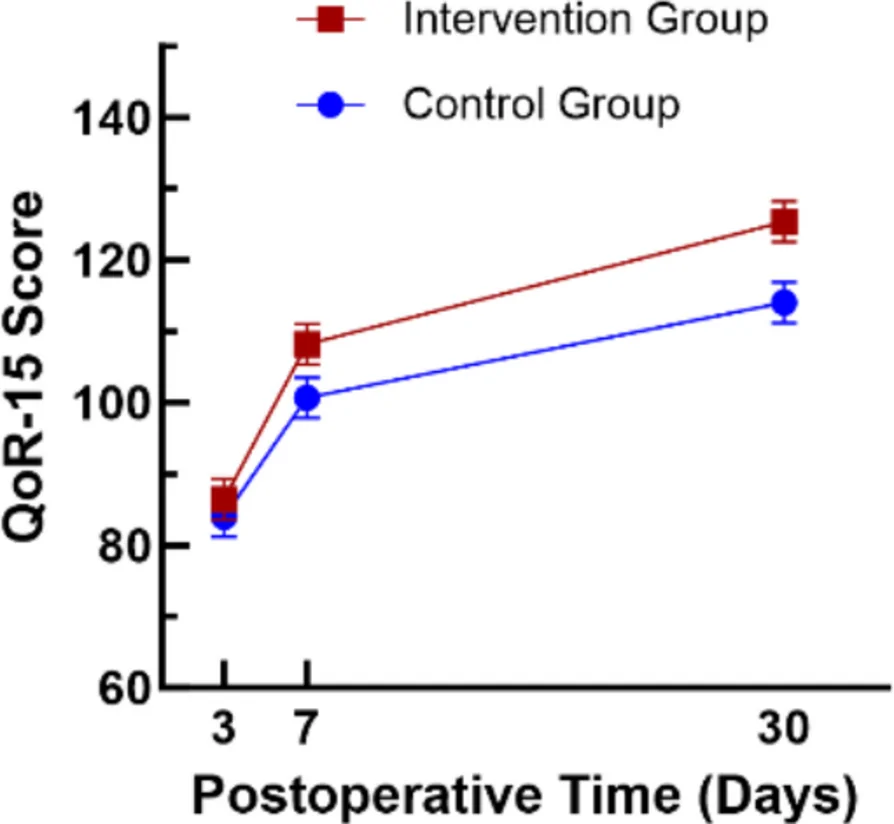
QoR-15 scores over time (EM mean ± SE**)**

Post hoc Bonferroni-adjusted comparisons showed no significant between-group differences at POD3 or POD7; however, QoR-15 scores were significantly higher in the intervention group at POD30 (mean difference = 11.33, 95% CI 3.41 to 19.26; *P* = 0.005), suggesting a delayed improvement in patient-reported recovery (Fig. 2).

### Secondary outcomes

**Fig. 3:**
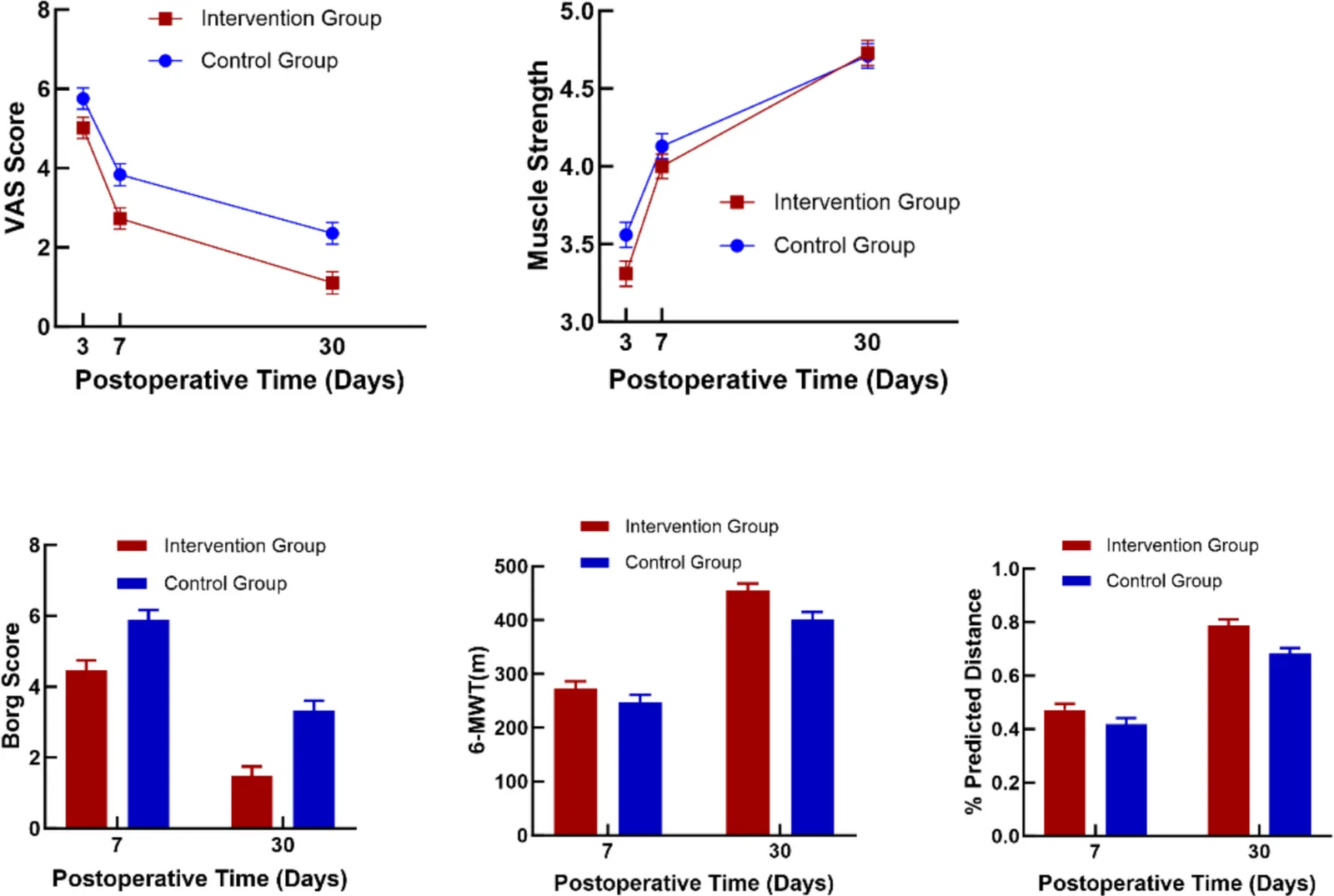
Secondary outcomes (EM mean ± SE). Estimated marginal means were derived from linear mixed-effects models

The detailed results of the secondary outcomes across different time points are summarized in Table 4 and Fig. 3.

**Table 4:** Comparison of Secondary Outcomes Between Groups Across Different Time Points

Outcome Measure	Time	Control (EM mean ± SE)	Intervention (EM mean ± SE)	Mean difference (95% CI)	*P* value
QoR-15	POD3	83.98 ± 2.83	86.42 ± 2.83	2.44 (− 5.48 to 10.37)	0.543
	POD7	100.67 ± 2.83	108.20 ± 2.83	7.53 (− 0.40 to 15.46)	0.062
	POD30	114.02 ± 2.83	125.36 ± 2.83	11.33 (3.41 to 19.26)	0.005
VAS Score	POD3	5.76 ± 0.27	5.02 ± 0.27	− 0.73 (− 1.49 to 0.02)	0.057
	POD7	3.84 ± 0.28	2.73 ± 0.28	− 1.11 (− 1.89 to − 0.33)	0.006
	POD30	2.36 ± 0.28	1.11 ± 0.28	− 1.24 (− 2.02 to − 0.47)	0.002
Muscle Strength	POD3	3.56 ± 0.08	3.31 ± 0.08	− 0.24 (− 0.48 to − 0.01)	0.039
	POD7	4.13 ± 0.08	4.00 ± 0.08	− 0.13 (− 0.37 to 0.10)	0.258
	POD30	4.71 ± 0.08	4.73 ± 0.08	0.02 (− 0.21 to 0.25)	0.850
Borg Score	POD7	5.89 ± 0.27	4.47 ± 0.27	− 1.42 (− 2.19 to − 0.66)	< 0.001
	POD30	3.34 ± 0.27	1.48 ± 0.27	− 1.87 (− 2.63 to − 1.10)	< 0.001
6MWT	POD7	247.29 ± 13.66	272.56 ± 13.66	25.27 (− 12.92 to 63.45)	0.193
	POD30	401.27 ± 13.66	454.60 ± 13.66	53.33 (15.15 to 91.52)	0.007
Proportion of predicted 6MWT distance	POD7	0.419 ± 0.022	0.473 ± 0.022	0.054 (− 0.007 to 0.115)	0.080
	POD30	0.682 ± 0.022	0.788 ± 0.022	0.106 (0.045 to 0.166)	0.001

Mean differences are presented as intervention minus control

Estimated marginal means and between-group differences were derived from linear mixed-effects models with Bonferroni-adjusted post hoc comparisons

#### Pain intensity (VAS scores)

Postoperative pain intensity assessed by VAS showed significant main effects of group (*P* = 0.003) and time (*P* < 0.001), with no significant group-by-time interaction (*P* = 0.465) in the linear mixed-effects model. Overall, VAS scores were lower in the intervention group. Between-group differences were not significant at POD3 but were significant at POD7 (mean difference = − 1.11, 95% CI − 1.89 to − 0.33; *P* = 0.006) and POD30 (mean difference = − 1.24, 95% CI − 2.02 to − 0.47; *P* = 0.002). Postoperative analgesic use during hospitalization was comparable between the two groups (Supplementary Table S1).

#### Muscle strength

The linear mixed-effects model for muscle strength showed a significant main effect of time (*P* < 0.001) but not group (*P* = 0.216). Interestingly, the intervention group exhibited slightly lower muscle strength than the control group at POD 3 (mean difference = − 0.24, 95% CI − 0.48 to − 0.01; *P* = 0.039). However, this transient difference dissipated, with no significant between-group differences observed at POD 7 or POD 30 (Table 4).

#### Borg scores

Perceived exertion assessed by the Borg score showed significant main effects of group (*P* < 0.001) and time (*P* < 0.001), with no significant group-by-time interaction (*P* = 0.089) in the linear mixed-effects model. Overall, Borg scores were lower in the intervention group. Between-group differences were significant at POD7 (mean difference = − 1.42, 95% CI − 2.19 to − 0.66; *P* < 0.001) and POD30 (mean difference = − 1.87, 95% CI − 2.63 to − 1.10; *P* < 0.001).

#### 6-Minute walk test

Functional capacity assessed by the six-minute walk test showed significant main effects of group (*P* = 0.021) and time (*P* < 0.001), with no significant group-by-time interaction (*P* = 0.154) in the linear mixed-effects model. Overall, 6MWT distance was greater in the intervention group. Between-group differences were not significant at POD7 but were significant at POD30 (mean difference = 53.33 m, 95% CI 15.15 to 91.52; *P* = 0.007).

#### Percentage of predicted 6MWT distance

The percentage of predicted 6MWT distance showed significant main effects of group (*P* = 0.003) and time (*P* < 0.001), with no significant group-by-time interaction (*P* = 0.128) in the linear mixed-effects model. Overall, the percentage of predicted 6MWT distance was higher in the intervention group. Between-group differences were not significant at POD7 but were significant at POD30 (mean difference = 0.106, 95% CI 0.045 to 0.166; *P* = 0.001).

### Adverse events

No serious adverse events related to the multimodal bundled intervention were reported during the study period. Among participants included in the modified intention-to-treat analysis, three individuals in the intervention group (6.7%) reported minor, transient discomfort associated with electroacupuncture; these events did not require additional intervention or lead to treatment discontinuation. No adverse events were reported in association with the abdominal massage or exercise therapy components.

## Discussion

In this single-center, single-blind pragmatic randomized controlled trial, a multimodal bundled intervention added to standard postoperative care did not significantly improve QoR-15 scores at POD3 or POD7. However, it was associated with a clinically meaningful improvement at POD30. Favorable differences in pain intensity, perceived exertion, and functional capacity were also observed, particularly at later postoperative assessments. While previous studies have reported beneficial effects of individual perioperative rehabilitation or adjunctive non-pharmacological interventions, evidence supporting structured real-world bundled interventions remains limited [25, 26]**.** These findings suggest a potential medium-term patient-reported and functional recovery benefit of this real-world bundled intervention; however, they do not demonstrate accelerated early ERAS-related recovery.

The absence of significant between-group differences in QoR-15 at POD3 and POD7 is clinically important. These time points represent the early postoperative phase most closely aligned with the objectives of ERAS pathways. Therefore, the present findings should not be interpreted as evidence that the intervention accelerated immediate postoperative recovery. One possible explanation for the POD30 difference is that early symptom-titrated rehabilitation may have contributed to gradual functional engagement after discharge. However, because physical activity, dietary intake, and analgesic use between POD7 and POD30 were not systematically recorded, this explanation remains hypothesis-generating.

The observed medium-term differences may be related to the combined effects of the intervention components, although the present pragmatic trial was not designed to isolate the contribution of each modality. Electroacupuncture and abdominal massage have been reported to modulate autonomic activity, inflammatory responses, and gastrointestinal motility [27–31]. More recent evidence suggests that electroacupuncture may ameliorate postoperative ileus through modulation of enteric glial cell activity and muscarinic receptor signaling [32]**,** while massage interventions may promote parasympathetic activation and physiological recovery [33]. Repeated acupoint stimulation may also reduce postoperative nausea, vomiting, and pain after laparoscopic surgery [34]. In addition, early mobilization is recognized as a key component of ERAS pathways and has been associated with improved postoperative recovery following gastrointestinal surgery [35]**.** In the present study, lower pain intensity and perceived exertion in the intervention group may have facilitated greater tolerance of progressive ambulation, which was reflected by better functional capacity at POD30. However, because detailed gastrointestinal recovery endpoints and post-discharge physical activity were not prospectively collected, and because complete dose-based analgesic consumption was unavailable, these mechanistic interpretations should be considered exploratory.

The functional findings provide an objective complement to the patient-reported QoR-15 results. QoR-15 was selected instead of QoR-40 because its shorter format reduces respondent burden while retaining multidimensional assessment of postoperative recovery, which was important for repeated assessments at POD3, POD7, and POD30 in this surgical oncology population. Although the QoR-15 is a validated and widely used patient-reported outcome measure for postoperative recovery, it remains partly subjective and may be influenced by pain perception, psychological status, expectations, and postoperative care experience. In this context, the observed improvement in 6MWT distance and the percentage of predicted 6MWT distance at POD30 supports the possibility that the POD30 QoR-15 difference was accompanied by improved functional recovery. Nevertheless, because preoperative QoR-15 and 6MWT measurements were not collected, we could not determine the extent to which patients returned to their individual preoperative recovery or functional status.

The pragmatic design of this trial should also be considered when interpreting the findings. The intervention was delivered as a bundled care program consisting of electroacupuncture, abdominal massage, structured breathing training, and symptom-titrated supervised ambulation, rather than as isolated components tested against sham controls. This design reflects real-world perioperative practice and allows evaluation of the overall effectiveness of a bundled intervention added to standard postoperative care. However, it also means that the independent contribution of each component cannot be determined. In addition, because participants and therapists could not be blinded, performance bias, placebo effects, and therapist attention effects cannot be fully excluded, particularly for patient-reported outcomes.

Several clinical implications can be considered. First, the intervention appeared feasible and safe in this postoperative surgical oncology population, with no serious intervention-related adverse events observed. Second, the symptom-titrated ambulation strategy may be more acceptable for older or frail postoperative patients than uniform activity targets, because progression was guided by pain and perceived exertion thresholds. Third, the findings suggest that integrated non-pharmacological rehabilitation may have value as an adjunct to standard ERAS pathways, particularly for medium-term patient-reported and functional recovery after discharge. However, given the lack of significant early QoR-15 differences at POD3 and POD7, the intervention should not be presented as a strategy that accelerates immediate postoperative recovery.

This study has several strengths. It used a randomized controlled design, blinded outcome assessment, and a patient-centered primary outcome. The intervention protocol was designed as a structured, multimodal, and symptom-titrated package that reflects real-world clinical practice. In addition, the use of both patient-reported and functional outcomes allowed a broader assessment of postoperative recovery than symptom scores alone.

This study has several important limitations. First, it was conducted at a single center with a modest sample size, which limits generalizability and reduces the ability to perform reliable subgroup analyses. Although age, frailty, tumor location, baseline functional status, and surgical procedure may plausibly modify the intervention effect, subgroup analyses were not prespecified in the protocol. We performed exploratory post hoc subgroup analyses using available variables, including age (< 70 vs ≥ 70 years) and preoperative hemoglobin level (≤ 110 vs > 110 g/L), but no statistically significant subgroup-by-treatment interactions were observed for QoR-15 or secondary outcomes. These findings should be interpreted cautiously because the analyses were underpowered, post hoc, and limited by modest subgroup sample sizes. Future multicenter trials should evaluate potential effect modification by age, frailty, tumor location, baseline functional status, and surgical procedure. Second, baseline preoperative QoR-15 and 6MWT measurements were not collected. Although additional preoperative clinical variables were retrospectively extracted to better characterize baseline comparability, the absence of baseline patient-reported and functional measurements prevents assessment of changes from baseline or recovery relative to each patient’s preoperative status.

Third, detailed postoperative analgesic consumption was not prospectively quantified. Although both groups received standardized multimodal analgesia under institutional ERAS protocols, the lack of complete dose-based analgesic data limits our ability to determine whether differences in pain scores and QoR-15 were independent of analgesic use. Fourth, the present trial was originally designed to evaluate patient-reported quality of recovery rather than gastrointestinal recovery as a prespecified endpoint. Therefore, detailed gastrointestinal recovery outcomes, such as time to first flatus, time to first defecation, tolerance of diet, postoperative ileus, and postoperative nausea or vomiting, were not systematically collected in a standardized manner. As a result, the potential effects of the multimodal bundled intervention on gastrointestinal recovery remain uncertain. Future trials should prospectively collect standardized gastrointestinal recovery indicators to better evaluate this aspect of postoperative recovery. Fifth, post-discharge factors between POD7 and POD30, including physical activity, dietary intake, rehabilitation adherence, and analgesic use, were not systematically recorded, which limits interpretation of the POD30 findings. Finally, because the intervention was a bundled pragmatic program, the specific contribution of electroacupuncture, abdominal massage, breathing training, and supervised ambulation cannot be separated.

Overall, this trial suggests that a multimodal bundled intervention may improve medium-term patient-reported and functional recovery after laparoscopic colorectal cancer surgery, but it did not improve early QoR-15 recovery during the ERAS-relevant period. The results should therefore be interpreted cautiously and viewed as supportive of further investigation rather than as definitive evidence for routine implementation. Larger multicenter randomized trials with baseline recovery and functional assessments, detailed analgesic and gastrointestinal recovery data, post-discharge monitoring, and prespecified subgroup analyses are needed to confirm these findings.

## Conclusions

In this single-center, single-blind pragmatic randomized controlled trial, a multimodal bundled intervention added to standard postoperative care did not significantly improve early QoR-15 scores at POD3 or POD7 in patients undergoing laparoscopic colorectal cancer surgery. However, the intervention was associated with a clinically meaningful improvement in QoR-15 at POD30, accompanied by favorable changes in pain intensity, perceived exertion, and functional capacity. These findings suggest a potential medium-term patient-reported and functional recovery benefit of this real-world bundled intervention, particularly after discharge, but they should not be interpreted as evidence of accelerated early ERAS-related recovery. Given the modest sample size, lack of baseline QoR-15 and 6MWT measurements, and limited data on post-discharge behavior and analgesic use, larger multicenter randomized trials with baseline recovery and functional assessments, detailed analgesic and gastrointestinal recovery data, post-discharge monitoring, and prespecified subgroup analyses are needed to confirm these findings.

## Supplementary Information


Supplementary Material 1.


## Data Availability

The datasets generated and/or analysed during the current study are not publicly available due to patient privacy and confidentiality, but are available from the corresponding author (Jiming Tao) on reasonable request.

## Abbreviations

6MWT: 6-Minute walk test
AJCC: American Joint Committee on Cancer
ASA: American Society of Anesthesiologists
BMI: Body mass index
CI: Confidence interval
CONSORT: Consolidated Standards of Reporting Trials
CRC: Colorectal cancer
EM mean: Estimated marginal means
ERAS: Enhanced Recovery After Surgery
LI-4: Hegu acupoint
MCID: Minimal clinically important difference
mITT: Modified intention-to-treat
MRC: Medical Research Council
POD: Postoperative day
PRO: Patient-reported outcome
QoR: Quality of Recovery
QoR-15: 15-Item Quality of Recovery
RCT: Randomized controlled trial
SD: Standard deviation
SE: Standard error
SNOSE: Sequentially numbered, opaque, sealed envelopes
ST-36: Zusanli acupoint
TEAS: Transcutaneous electrical acupoint stimulation
VAS: Visual Analog Scale
