# Supplementary material for: Effect of a multimodal bundled intervention on quality of recovery after laparoscopic colorectal cancer surgery: a single-center, single-blind, pragmatic randomized controlled trial
Source: World J Surg Oncol. 2026 Jun 17;24:353. doi: 10.1186/s12957-026-04454-9 (PMC13531738; doi:10.1186/s12957-026-04454-9)
Supplement: Supplementary file 1 [file 12957_2026_4454_MOESM1_ESM.pdf]

Supplementary Table S1. Postoperative analgesic use during hospitalization

Analgesic variable	Control group (n = 45)	Intervention group (n = 45)	P value
PCA use, n (%)	37 (82.2)	41 (91.1)	0.353
Any opioid use, n (%)	39 (86.7)	42 (93.3)	0.485
Any non-opioid analgesic use, n (%)	14 (31.1)	18 (40.0)	0.509
Patients requiring rescue analgesia, n (%)	18 (40.0)	23 (51.1)	0.397
Rescue analgesic administrations, median (IQR)	0 (0, 1)	1 (0, 1)	0.353

Data are presented as n (%) or median (IQR), as appropriate. Percentages were calculated using n = 45 per group. Binary variables were compared using Fisher's exact test, and rescue analgesic administrations were compared using the Mann–Whitney U test. Rescue analgesic administrations were counted from the day of surgery to hospital discharge.

Supplementary Table S2. Exploratory subgroup analyses by age and preoperative hemoglobin level

Outcome	Model/assessment	Age subgroup interaction P value	Hemoglobin subgroup interaction P value
QoR-15	Trajectory across POD3, POD7, and POD30	0.237	0.383
Pain intensity (VAS)	Trajectory across POD3, POD7, and POD30	0.913	0.098
Muscle strength	Trajectory across POD3, POD7, and POD30	0.402	0.658
Borg score	Trajectory across POD7 and POD30	0.545	0.349
Walking distance	Trajectory across POD7 and POD30	0.666	0.895
Percentage of predicted 6MWT distance	Trajectory across POD7 and POD30	0.483	0.562
6MWT distance	POD30 assessment	0.290	0.138

Values are P values for subgroup-by-treatment interaction terms. Age was dichotomized as <70 versus≥70 years. Preoperative hemoglobin was dichotomized as≤110 versus >110 g/L. For repeated outcomes, interaction effects were assessed using mixed-effects models including treatment group,

time, subgroup, and their interaction terms. For POD30 6MWT distance, interaction effects were assessed using a linear model including treatment group, subgroup, and their interaction term. These analyses were exploratory and not prespecified.
